# Vascular endothelial growth factor attenuates neointimal hyperplasia of decellularized small-diameter vascular grafts by modulating the local inflammatory response

**DOI:** 10.3389/fbioe.2022.1066266

**Published:** 2022-12-20

**Authors:** Xinlong Xie, Qiying Wu, Yuhong Liu, Chunyang Chen, Zeguo Chen, Chao Xie, Mingzhe Song, Zhenlin Jiang, Xiaoke Qi, Sixi Liu, Zhenjie Tang, Zhongshi Wu

**Affiliations:** ^1^ Department of Cardiovascular Surgery, The Second Xiangya Hospital, Central South University, Changsha, Hunan, China; ^2^ Department of Cardiothoracic Surgery, The First Affiliated Hospital, Hunan University of Chinese Medicine, Changsha, Hunan, China; ^3^ Engineering Laboratory of Hunan Province for Cardiovascular Biomaterials, Changsha, Hunan, China

**Keywords:** neointimal hyperplasia, macrophages, vascular endothelial growth factor, small-diameter vascular graft, decellularized

## Abstract

Small-diameter vascular grafts (diameter <6 mm) are in high demand in clinical practice. Neointimal hyperplasia, a common complication after implantation of small-diameter vascular grafts, is one of the common causes of graft failure. Modulation of local inflammatory responses is a promising strategy to attenuates neointimal hyperplasia. Vascular endothelial growth factor (VEGF) is an angiogenesis stimulator that also induces macrophage polarization and modulates inflammatory responses. In the present study, we evaluated the effect of VEGF on the neointima hyperplasia and local inflammatory responses of decellularized vascular grafts. In the presence of rhVEGF-165 in RAW264.6 macrophage culture, rhVEGF-165 induces RAW264.6 macrophage polarization to M2 phenotype. Decellularized bovine internal mammary arteries were implanted into the subcutaneous and infrarenal abdominal aorta of New Zealand rabbits, with rhVEGF-165 applied locally to the adventitial of the grafts. The vascular grafts were removed en-bloc and submitted to histological and immunofluorescence analyses on days 7 and 28 following implantation. The thickness of the fibrous capsule and neointima was thinner in the VEGF group than that in the control group. In the immunofluorescence analysis, the number of M2 macrophages and the ratio of M2/M1 macrophages in vascular grafts in the VEGF group were higher than those in the control group, and the proinflammatory factor IL-1 was expressed less than in the control group, but the anti-inflammatory factor IL-10 was expressed more. In conclusion, local VEGF administration attenuates neointimal hyperplasia in decellularized small-diameter vascular grafts by inducing macrophage M2 polarization and modulating the inflammatory response.

## 1 Introduction

Cardiovascular disease (CVD) is the leading cause of mortality and morbidity worldwide, accounting for about one-third of all deaths each year (Weekes et al., 2022). Coronary artery diseases (CAD) is the most common type of CVD and coronary artery bypass surgery (CABG) remains the preferred treatment. Currently, autologous blood vessels (such as an internal mammary artery and saphenous vein) are the gold standard grafting materials for CABG surgery. However, these grafts are not always available due to limited quantity and length and poor quality (Kajbafzadeh et al., 2017).

Non-biodegradable synthetic grafts such as expanded polytetrafluoroethylene (ePTFE) have achieved remarkable success in large diameter vessels (diameter >6 mm) but have failed in small diameter vessels (diameter <6 mm) due to low patency, thrombosis, neointimal hyperplasia and lack of growth potential (Desai et al., 2011; Drews et al., 2017). Biodegradable synthetic grafts such as poly (lactic acid) (PLA), poly (glycolic acid) (PGA) and polycaprolactone (PCL) can be degraded into non-toxic components *in vivo* with controllable degradation rate. However, they also have many disadvantages such as loss of mechanical strength in the early stages of implantation, calcification, and neointimal hyperplasia (de Valence et al., 2012; Radke et al., 2018). Decellularized extracellular matrix (ECM) scaffolds are extensively used as biomaterials due to its natural tissue architecture, components, biomechanical properties and functional bioactive molecules required for tissue regeneration. Therefore, small-diameter vascular grafts based on decellularized extracellular matrix have attracted the attention of more and more researchers (Ilanlou et al., 2019). However, the neointimal hyperplasia limits its clinical application (Yang et al., 2019; Lopera Higuita and Griffiths, 2020b).

Neointimal hyperplasia is a major cause of failure of small-diameter vascular grafts after implantation (Reinhardt. et al., 2019; Lopera Higuita and Griffiths, 2020a; Yu et al., 2021). The development of the neointima involves interactions between serum proteins, platelets, inflammatory proteins/cells, fibroblasts/smooth muscle cell -like cells and endothelial cells (Reinhardt. et al., 2019). There are different strategies to inhibit neointimal hyperplasia targeting various elements involved in the process of neointimal formation, and the repolarization of macrophages to an anti-inflammatory M2 phenotype and positive regulation of the local inflammatory environment may be a promising strategy (Tan et al., 2019).

Vascular endothelial growth factor (VEGF) is a key player in angiogenesis and is therefore widely used to stimulate endothelialization of vascular grafts (Zhang et al., 2013; Koobatian et al., 2016; Kong et al., 2019). Recently, Wheeler et al. found that both endogenous decidual VEGF and exogenous recombinant VEGF could stimulate macrophage migration and polarization towards the M2 phenotype in HTP-1 cell culture (Wheeler et al., 2018). Smith et al. found that in VEGF-containing grafts, infiltrating monocytes transformed into anti-inflammatory M2 phenotype macrophages and the grafts formed a well-defined lumen and medial layer similar to those of natural arteries. VEGF creates an anti-inflammatory microenvironment that promotes regeneration of decellularized vascular grafts (Smith et al., 2019). Therefore, we hypothesized that VEGF may attenuates neointimal hyperplasia in vascular grafts by inducing macrophage polarization towards M2 phenotype and modulating local inflammatory responses.

**FIGURE 1:**
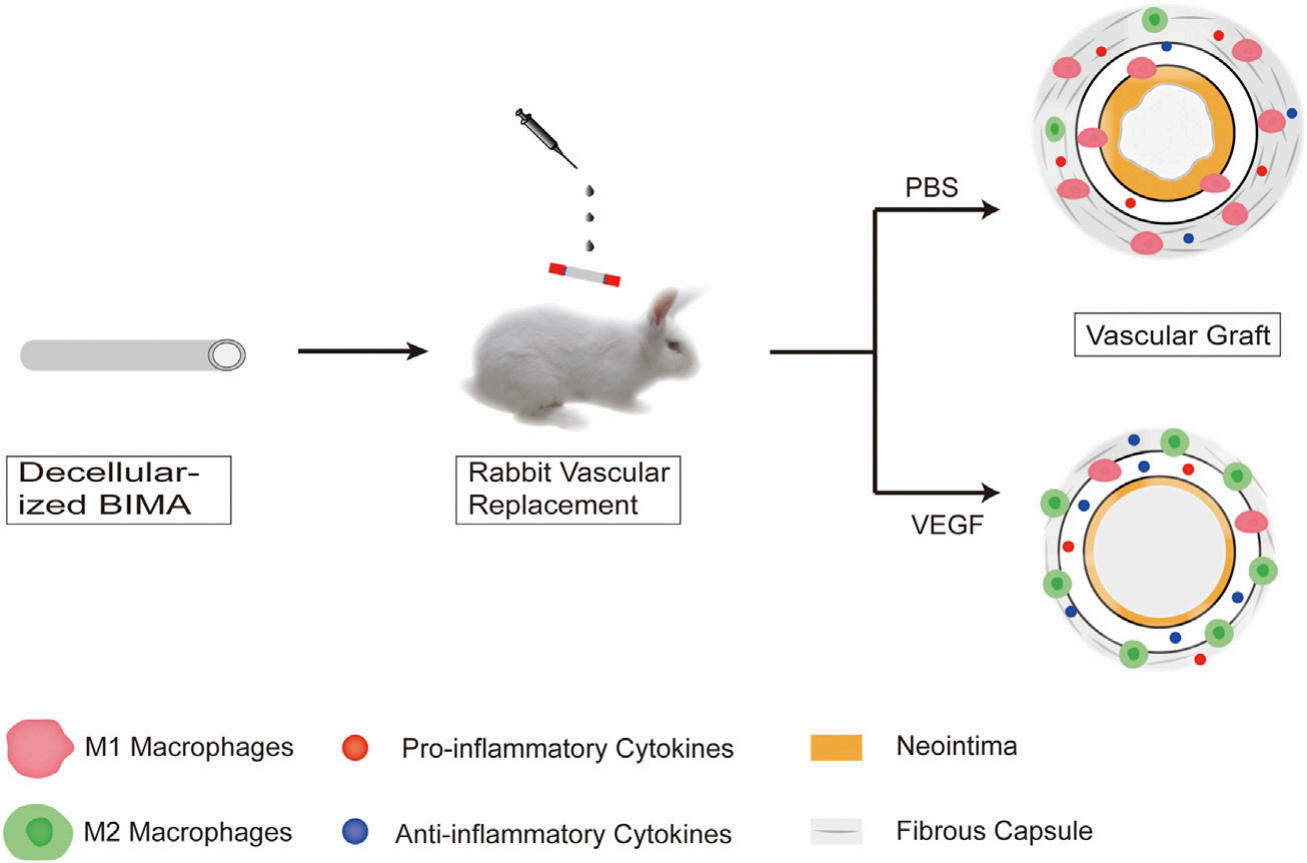
Schematic overview of the overall study.

In this study, we successfully produced decellularized bovine internal mammary arteries (BIMAs) which have a desired length (15–20 cm) and diameter (3–5 mm) (Liu et al., 2022) and assessed the influence of our decellularization approach on their biochemical composition and biomechanical properties. In RAW264.7 cell cultures, we demonstrated that VEGF induces macrophage polarization towards the M2 phenotype. Furthermore, immunofluorescence and histological analyses of subcutaneous and abdominal aortic graft implants from New Zealand rabbits were used to quantify macrophage phenotype, local cytokine expression, and fibrous capsule and neointimal thickness (Figure 1).

## 2 Materials and methods

### 2.1 BIMAs harvesting

Fresh BIMAs were obtained from a local slaughterhouse (warm ischemic time<0.5 h) under clean conditions and transferred to the laboratory in phosphate-buffered solution (PBS). After the excision of perivascular connective tissue, BIMAs were stored at 4°C in PBS supplemented with 1% streptomycin/penicillin until processing.

### 2.2 Decellularization

The BIMAs were cut into a length of 10 cm for decellularization. First, following connection to a peristaltic pump, BIMAs were washed serially with a pump flow at 50 ml/min while shaking at 120 rpm with detergents (0.5% Sodium Dodecyl Sulfate (SDS) and 0.5% Sodium Deoxycholate (SD) in 10 mM Tris-Hcl buffer (pH 8.0) for 24 h at room temperature. Subsequently, BIMAs underwent 24 h of ribozyme digestion (45 U/ml DNase-I and 0.3 mg/ml RNase in 10 mM Tris–HCl buffer, pH 7.5) at 37°C. Finally, BIMAs were treated with shaking (120 rpm) in pH 7.5 10 mM Tris-Hcl buffer at room temperature for 48 h. After each step, BIMAs were rinsed with saline 3 times for 10 min each time. All solutions contains 1% penicillin—streptomycin and were changed twice daily. Decellularized BIMAs were preserved in 60% alcohol after sterilization by gamma radiation at a dose of 25 KGy.

### 2.3 Histology

Both native and decellularized specimens were fixed in 4% paraformaldehyde solution for 24 h at room temperature. The specimens were then dehydrated in graded ethanol, paraffin-embedded and cut to 5 μm thickness. Hematoxylin and eosin (H&E) and 4′,6-diamidino-2-phenylindole (DAPI) staining were performed to evaluate decellularization efficiency and gross tissue morphology. Moreover, the preservation of collagen and elastic fibers were evaluated by Masson’s trichrome and elastic Van-Gieson (EVG) staining methods, respectively.

### 2.4 DNA and biochemistry quantification

Both native and decellularized specimens were immersed in the TE buffer to extract total DNA with the Genomic DNA purification Kit (TIANamp Genomic DNA kit). The concentration of DNA in the solution was quantified by spectrophotometry using NanoDrop Technology (Thermo scientific Nanodrop 1,000).

The ECM components collagens and elastin were quantified by means of soluble collagen assay (Nanjing Jiancheng Co., Ltd. China) and Fastin elastin assay kits (Biocolor Ltd., United Kingdom) according to the manufacturer’s protocol.

### 2.5 Mechanical properties

Uniaxial tensile tests were performed on native and decellularized BIMAs using an INSTRON testing machine to evaluate the effect of decellularization on Young’s modulus, ultimate tensile stress and suture strength. All specimens were cut to about 3 cm length along the long axis and loaded on the testing device with regular length (the distance between sample holders, 1 cm) and subsequently tested in the longitudinal-axial direction at room temperature with an extension speed of 10 mm/min until failure. Suture retention strength was measured using a 6–0 prolene suture placed on one side of the artery 2 mm from the edge. The burst pressure of native and decellularized BIMAs were measured by delivering PBS from a syringe *via* a control valve, and the maximum pressure before rupture was recorded as the burst pressure.

### 2.6 RAW 264.7 cell culture

RAW264.7 macrophages were purchased from the Procell Life Science&Technology Co. (China, Wuhan) and cultured in a humidified incubator containing 95% air and 5% CO_2_ at 37°C in Dulbecco’s Modified Eagle Medium (DMEM) supplemented with 10% fetal bovine serum (FBS), penicillin (100 U/mL) and streptomycin (100 μg/ml).

### 2.7 Cell viability assay

Cell Counting Kit-8 (CCK-8) reagent can be used for cell proliferation and toxicity analysis (Wang et al., 2018). The *in vitro* cytotoxicity of different concentrations of rhVEGF-165(Cell Signaling Technology, United States) (0,10, 50, 100 ng/ml) was determined by the CCK-8 assay. Briefly, RAW264.7 macrophages were seeded in 96-well plates at a density of 5 × 10^3^ cells/well. After 24 h, the medium was changed to contain different concentrations of rhVEGF-165. A further 24 h later, 10 μl of CCK-8 solution was added to each well and incubated for another 2 h. Finally, the optical density (OD) at 450 was measured using spectrophotometer (Thermo scientific 1530, Singapore).

### 2.8 *In vitro* macrophage polarization assay

RAW264.7 macrophages were seeded into a 6-well plate at a density of 5× 10^5^ cells/well and grown in DMEM (total volume, 2,000 μl). When the cells reached 60% confluence, the medium was removed, and the cells were incubated with 100 ng/ml Lipopolysaccharides (LPS, HY-d1056, Medchemical, China), 10 ng/ml Interlekin-4 (IL-4, HY-P7080, Medchemical, China) and different concentrations of rhVEGF-165 (10, 50, and 100 ng/ml) (Cell Signaling Technology, United States) in medium for 24 h. To assess the polarization of macrophages, the expression of phenotypic markers was detected by flow cytometry. Briefly, RAW264.7 macrophages were treated with cold PBS and 4 × 10^5^ cells were counted and suspended in 100 μl of PBS supplemented with 5% FBS. Cells were then incubated with anti-CD11b (1:100; APC-65055, Proteintech, China), anti-CD86 (1:100, FITC-65068, Proteintech, China) and anti-CD206 (1:200, 18704-1-AP, Proteintech, China) at 4°C in the dark for 30 min. After three washes with PBS, the labeled cells were suspended in 500 µl PBS, and data were acquired by FACSCantoll cytometer system (BD, United States) and analyzed using FlowJo software.

We found that 50 ng/ml rhVEGF-165 stimulated the M2 polarization of RAW264.7 macrophages with the best efficiency. Therefore, we stimulated RAW264.7 macrophages with PBS, LPS, IL-4 and 50 ng/ml rhVEGF-165 as above for the following experiments.

To determine the expression levels of M1-related factors (IL-1β and TNF-α) and M2-related factors (IL-10 and CD206), treated cellular mRNA was extracted with Total RNA Extraction Reagent (GeneJET RNA Purification Kit, thermoscientific, United States) according to the manufacturer’s instructions. Subsequently, 1 μg of total RNA was reverse transcribed to cDNA using Reverse Transcription Master Mix (Maxima H Minus First Strand cDNA Synthesis Kit with dsDNase, thermoscientific, United States). Finally, qRT-PCR was performed using SYBR Green qPCR Master Mix. All experiments were performed in triplicate and data were normalized for HPRT. Relative expression levels were calculated by calculated using the 2^−ΔΔCq^ method. Primers for qRT-PCR are listed in Table 1.

**TABLE 1:** Primers for quantitative real-time PCR.

Gene		Primer
M1	TNF-α	F 5′-GGA​CTA​GCC​AGG​AGG​GAG​AAC​AG-3′
		R 5′-GCC​ACA​AGC​AGG​AAT​GAG​AAG​AGG-3′
	IL-1β	F 5′-TTC​AGG​CAG​GCA​GTA​TCA​CTC​ATT​G-3′
		R 5′-TGT​CGT​TGC​TTG​GTT​CTC​CTT​GTA​C-3′
M2	CD206	F 5′-CAA​GCG​ATG​TGC​CTA​CC-3′
		R 5′-AAT​GCT​GTG​GAT​ACT​TGC​C-3′
	Il-10	F 5′-GCC​CTT​TGC​TAT​GGT​GTC-3′
		R 5′-TCT​CCC​TGG​TTT​CTC​TTC​C-3′
HPRT		F 5′-TAC​AGG​CCA​GAC​TTT​GTT​GGA-3′
		R 5′-ACT​TGC​GCT​CAT​CTT​AGG​CT-3′

### 2.9 Animals

All animal experiments were conducted in accordance with the guidelines for animal experiments established by Institutional Animal Care and Use Committee (IACUC) of Second Xiangya Hospital of Central South University. Female adult New Zealand rabbits (3,000–3,500 g of body weight) were used for subcutaneous implantation and abdominal artery implantation of decellularized small-diameter vascular grafts.

### 2.10 Rabbit subcutaneous implantation

Grafts of two groups (Control/VEGF, n = 5 per group per time point) were evaluated in a total of 20 rabbits. Animals were anesthetized and sedated with sodium pentobarbital at 30 mg kg^−1^. The back portion of the animal was shaved, a 2.0 cm skin incision was made and a 2 × 2 cm pockets were created, one on each side of the spine. A 1 × 1 cm piece of decellularized BIMAs vascular sheet was placed in each rabbit in two pockets, with the adventitial side of each vascular sheet facing the skin. For local VEGF application, 100 ng rhVEGF-165 dissolved in PBS (concentration,2 ng/μL; total volume, 50 μl) was dropped evenly on the adventitia of decellularized vascular sheets in the VEGF group. The control group was given an equal volume of PBS in the same way. Then, the incision closed routinely. At days 7 and 28 after implantation, rabbits were euthanized and specimens were excised en-bloc.

### 2.11 Rabbit abdominal artery implantation

Grafts of two groups (Control/VEGF, n = 5 per group per time point) were evaluated in a total of 20 rabbits. Animals were anesthetized and sedated with sodium pentobarbital at 30 mg kg^−1^. The left abdomen portion of the animal was shaved, and the infrarenal abdominal aorta was dissected free from surrounding tissues after a left flank abdominal incision. Heparin (1 mg/kg) was injected intravenously and the proximal and distal ends of the artery at the implantation site were blocked with non-invasive hemostasis clips. Decellularized vascular grafts (length 2 cm, diameter 4 mm) were anastomosed to the infrarenal abdominal aorta in an end-to-end manner with 7–0 prolene suture. For local VEGF application, rhVEGF-165 (dosage, 100 ng/cm ^2^) dissolved in PBS (concentration, 2 ng/μl; total volume, approximately 125 μl) was dropped evenly on the adventitia of decellularized vascular grafts in the VEGF group. The decellularized group was given an equal volume of PBS in the same way. To prevent the risk of graft thrombosis, animals received low-molecular-weight heparin calcium (60 IU kg^−1^) treatment until 1 week after surgery. Graft patency was monitored by color Doppler ultrasound on days 7 and 28 post-implantation, and then the graft was excised en-bloc.

### 2.12 Histology and immunofluorescence

All specimens removed from New Zealand rabbits were fixed in 4% paraformaldehyde for 24 h and embedded in paraffin for histology. The specimens were sliced (5 μm) and stained with HE. The thickness of the fibrous capsule and vascular neointima was assessed by quantifying histological sections of each specimen using NIS-Elements D software. To assess thickness of the fibrous capsule and vascular neointima, 5 HE staining images were used per tissue section for each animal (n = 5 animals, per group per time point) for a total of 25 images for per index.

For immunofluorescence staining, total macrophages were stained with mouse anti-CD68 primary antibody (1:200, GB14043, Servicebio, China). M1 macrophages were stained with mouse anti-CD86 primary antibody (1:100, ab220188, Abcam, United States). M2 macrophages were stained with mouse anti-CD206 primary antibody (1:200, 60143-1-lg, Proteintech, China). Pro-inflammatory cytokine Interleukin-1β (IL-1β) was stained with mouse anti-IL-1β primary antibody (1:400, 66737-1-lg, Proteintech, China). Anti-inflammatory cytokine Interleukin- 10 (IL-10) was stained with mouse anti-IL-10 primary antibody (1:1,000, GB12108, Servicebio, China). Endothelial cells were stained with mouse anti-eNOS primary antibody (1:1,000, Ab76198, abcam, United States). Smooth muscle cells were stained with mouse anti-α-SMA primary antibody (1:40, GTX18147, GeneTex, United States) and mouse anti-Calponin primary antibody (1:200, C2687, Sigma, United States) respectively. Then Alexa Flour 488 anti-goat immunoglobulin G secondary antibody (1:300, GB25301, Servicebio, China) and CY3 anti-goat immunoglobulin G secondary antibody (1:300, GB21301, Servicebio, China) were used, respectively. Nuclei were stained with DAPI. Five images were used per tissue section for each animal (n = 5 animals, per group per time point) for a total of 25 images per antibody panel.

### 2.13 Statistical analysis

All data are expressed as mean ± standard error of the mean (SEM). The normal distribution of our data sets was confirmed using the Kolmogorov-Smirnov test and parametric analysis was performed. One-way analysis of variance and unpaired Student’s t-test were used to determine the significant differences among the groups, and *p*-values <0.05 was considered significant.

## 3 Results

### 3.1 Bovine internal mammary arteries are successfully decellularized while preserving extracellular matrix components and mechanical properties

**FIGURE 2:**
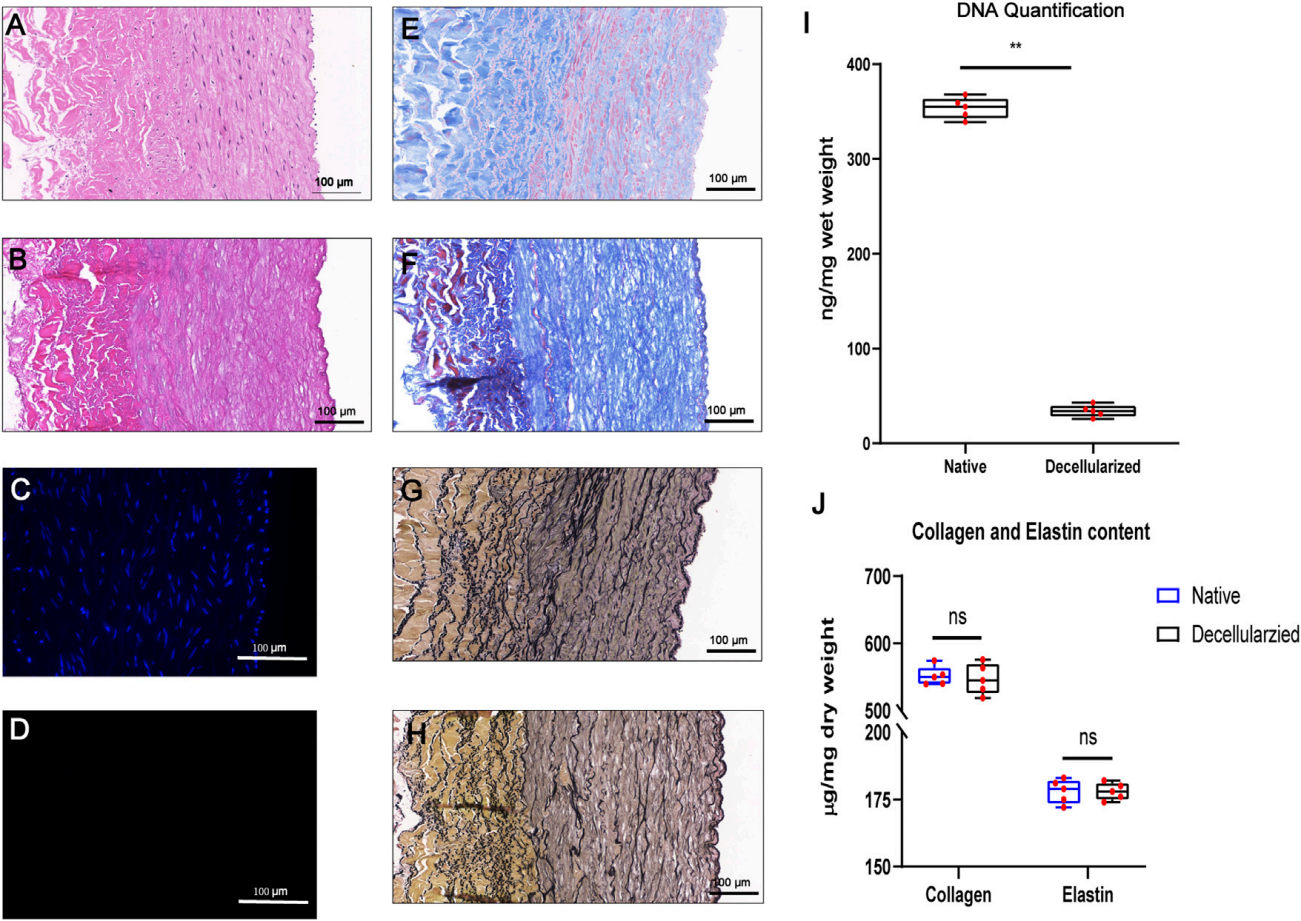
Successful decellularization of bovine internal mammary artery (BIMA) while preserving extracellular matrix components. Characteristics of the bovine internal mammary artery (BIMA) before **(A,C,E, and G)** and after **(B,D,F, and H)** decellularization. H&E staining **(A,B)**, DAPI staining **(C,D)** Masson’s trichrome staining **(E,F)** and elastic Van-Gieson staining **(G,H)**. DNA quantification of native and decellularized BIMA **(I)**. Content of collagen and elastin was insignificant between native and decellularized BIMA **(J)**. *p < 0.05. **p < 0.01. ns represents no significant difference.

To initially evaluate BIMAs for tissue engineering, we established a decellularization protocol and evaluated the decellularization effect. HE and DAPI staining revealed a complete removal of cellular and nucleic components in the BIMAs after decellularization procedure (Figures 2A–D). DNA quantification confirmed a significant reduction of DNA content in decellularized BIMAs compared to native BIMAs (Figure 2I). Masson’s trichrome staining and EVG staining showed that collagen and elastic fibers were well preserved, respectively (Figures 2E–H). The hydroxyproline and elastin assay revealed there were no difference in elastin and collagen content between decellularized and native BIMAs (Figure 2J).

**FIGURE 3:**
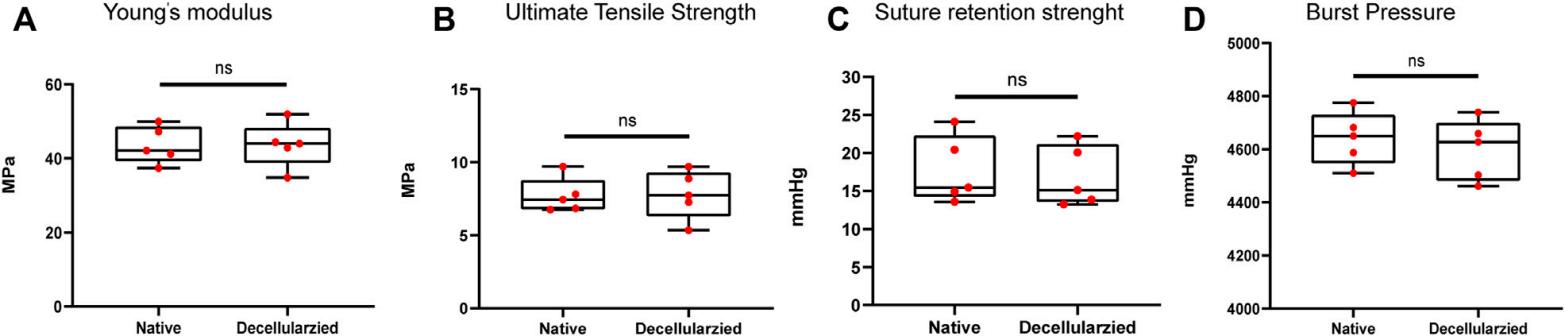
Full preservation of the mechanical properties of bovine internal mammary artery (BIMA) after decellularization. **(A)** Young’s modulus. **(B)** Ultimate Tensile Strength. **(C)** Suture retention strength. **(D)** Burst pressure. *p < 0.05. **p < 0.01. ns represents no significant difference.

The parameters of Young’s modulus, Ultimate tensile strength, burst pressure and suture retention strength showed no significant change in mechanical properties between native and decellularized BIMAs (Figures 3A–D).

### 3.2 VEGF induces macrophage polarization *in vitro*

**FIGURE 4:**
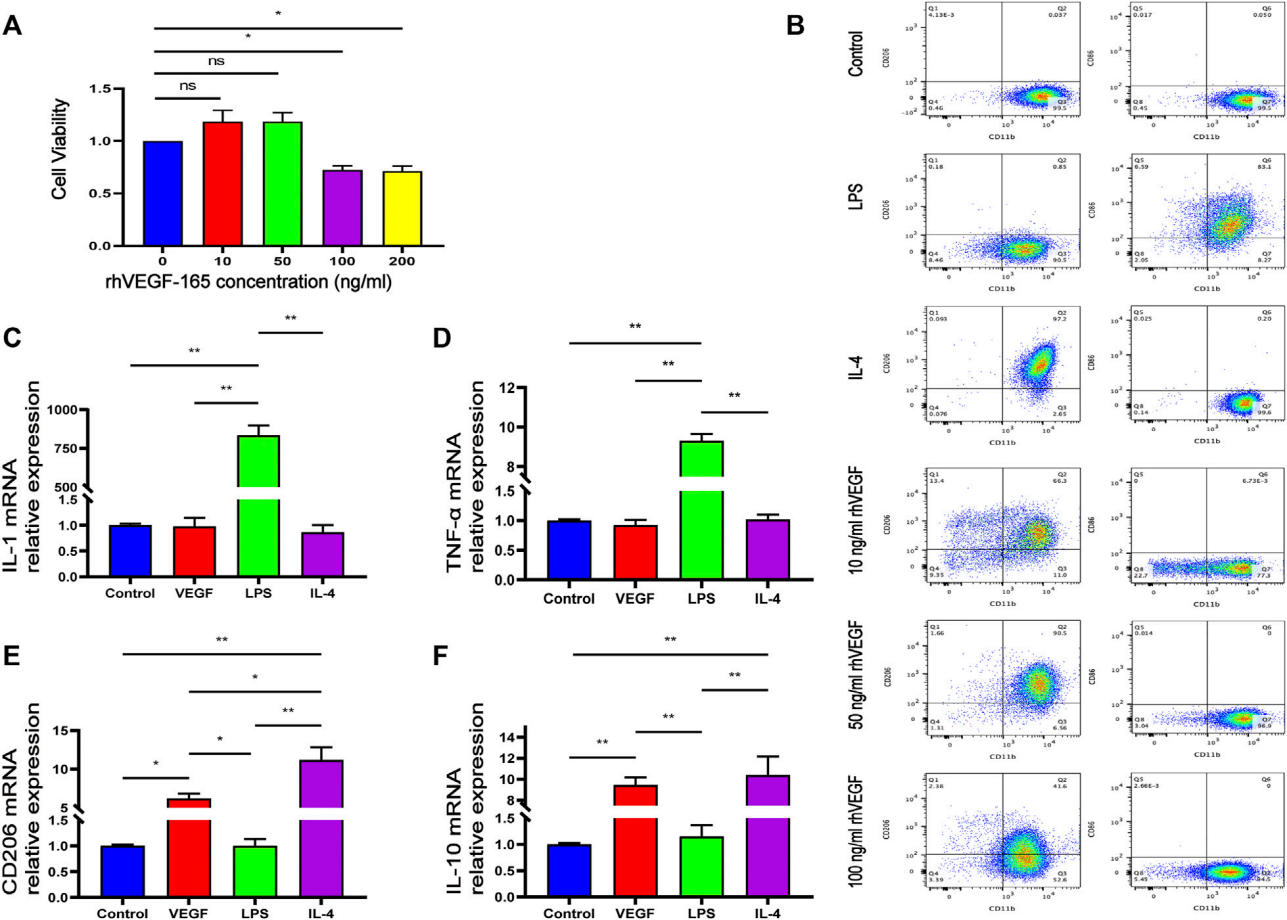
Viability of RAW264.7 macrophages after 24 h of incubation with different concentrations of rhVEGF-165 **(A)**. The proportion of M1 to M2 macrophages in RAW264.7 cells in each group **(B)**. The expression levels of M1 or M2 macrophage markers in RAW264.7 cells in each group **(C,D,E and F)**. *p < 0.05. **p < 0.01. ns represents no significant difference.

In order to evaluate the *in vitro* cytotoxicity of rhVEGF-165 in RAW264.7 macrophages, macrophages were incubated with rhVEGFF-165 for 24 h, and cell viability was detected by CCK-8 assay. As shown in Figure 4A, rhVEGF-165 was not toxic to RAW264.7 macrophages at concentrations up to 50 ng/ml. However, when macrophages were treated with rhVEGF-165 at concentrations up to and above 100 ng/ml, a significant inhibition of cell growth was observed.

To investigate the effect of rhVEGF-165 on macrophage polarization, flow cytometry was used to evaluate the expression of the M0 marker CD11b, the M1 marker CD86 and the M2 marker CD206 in RAW264.7 macrophages. Flow cytometry analysis showed that LPS significantly stimulated the polarization of M0 macrophages to M1 (CD86 positive), and IL-4 significantly stimulated the polarization of M0 macrophages to M2 (CD206 positive), consistent with previous reports (Zhang et al., 2021). With different concentrations of rhVEGF-165 stimulation, we detected different proportions of M2 macrophages and the highest proportion of M2 after treated with 50 ng/ml rhVEGF-165 (Figure 4B).

Quantitative real-time PCR was used to measure the mRNA expression of the M1 (IL-1β and TNF-α) and M2 (CD206 and IL-10) phenotype genes. The expression levels of IL-1β and TNF-α in rhVEGF-165-treated RAW 264.7 macrophages were significantly decreased, while the expression levels of CD206 and IL-10 were significantly increased, compared with LPS-treated RAW 264.7 macrophages (Figures 4C–F). These results suggest that VEGF polarizes RAW 264.7 macrophages toward an M2 phenotype *in vitro*.

### 3.3 VEGF reduces fibrous capsule formation in subcutaneous implants

**FIGURE 5:**
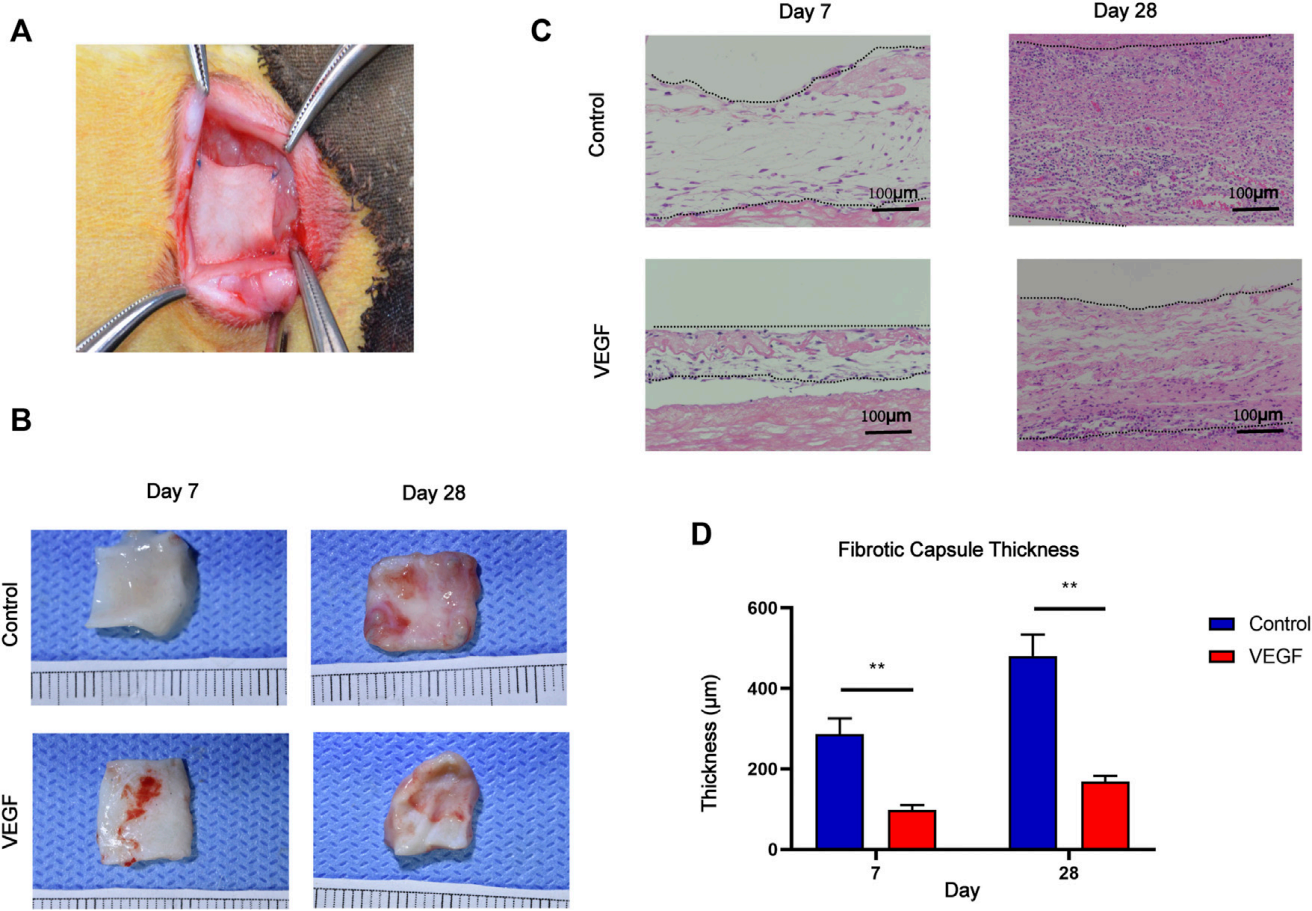
Macroscopic images of the rabbit subcutaneous implantation model **(A)**. Gross images of subcutaneous implants at days 7 and 28 in each group **(B)**. HE staining of subcutaneous implants at days 7 and 28 in each group. Between the two dashed lines depicts the fibrous capsule of subcutaneous implants **(C)**. Measurement of fibrous capsule thickness **(D)**. *p < 0.05. **p < 0.01. ns represents no significant difference.

**FIGURE 6:**
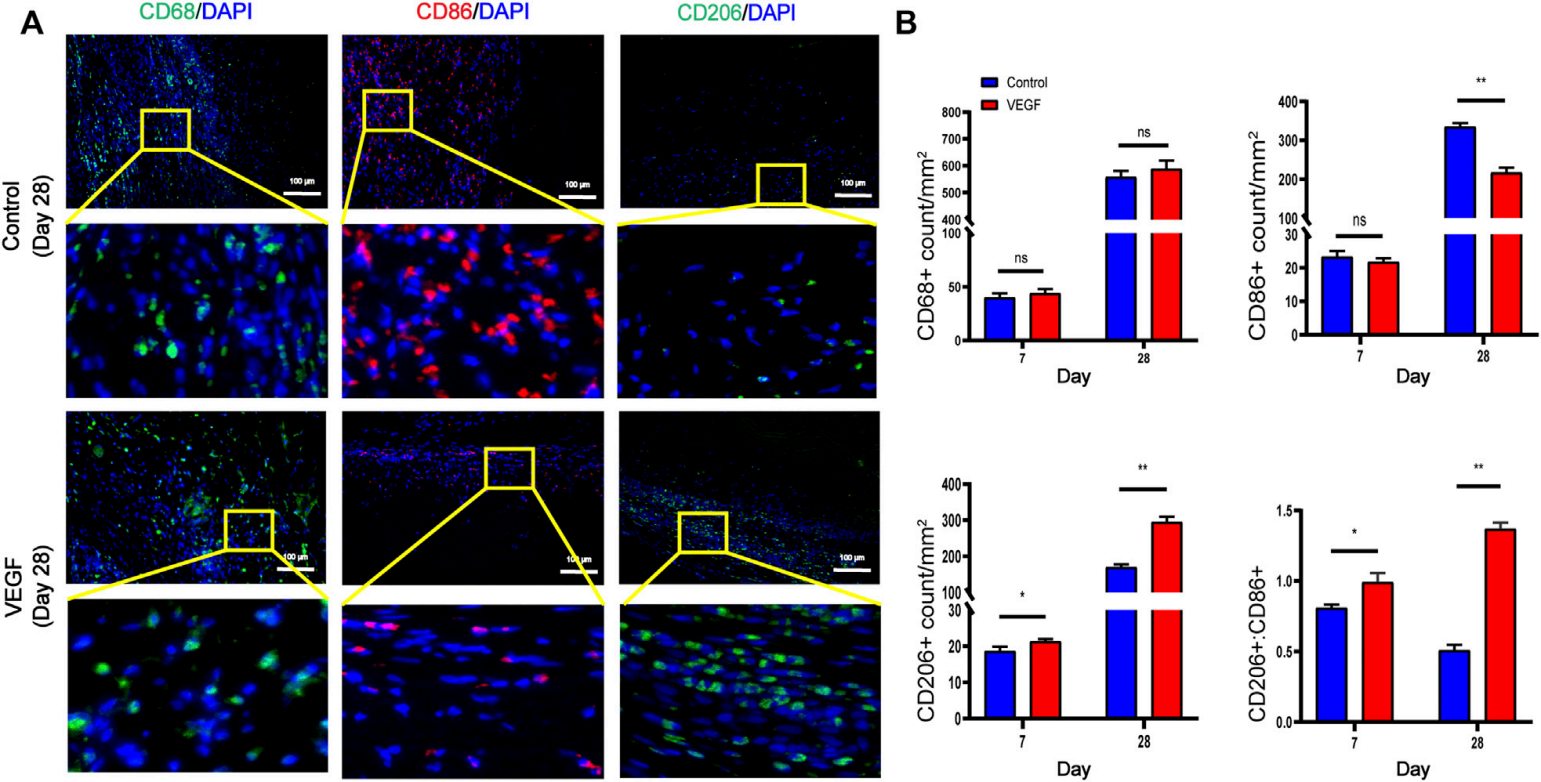
Representative immunofluorescence images of subcutaneous implants at day 28 in each group **(A)**. CD68 and CD206 stained in green, CD86 stained in red. Quantification of CD68^+^ total macrophages, CD86^+^ M1-macrophages, CD206+ M2-macrophages and M2/M1 (CD206+/CD86+) ratio **(B)**. *p < 0.05. **p < 0.01. ns represents no significant difference.

The New Zealand rabbit subcutaneous implantation model was used to assess the *in vivo* function of rhVEGF-165 (Figure 5A), which was then further assessed in the vascular environment. Both on visual inspection and HE staining, we observed that the fibrous capsule was thinner in the VEGF group than in the control group (Figures 5B, C). The fibrotic capsule thickness of the control group increased over time from 286.15 ± 39.64 μm at day 7 to 528.88 ± 66.01 μm at day 28 (Figure 5D). In contrast, the fibrous capsule thickness of the VEGF group was reduced at all time points compared with the control group (Figure 5D). Immunostaining markers of total macrophage (CD68), M1 (CD86), and M2 (CD206) phenotypes were used to assess macrophage response. The VEGF group had a higher density of CD206-positive cells at days 7 and 28 compared to the control group (Day 7,21.41 ± 0.57 count/mm^2^ vs. 18.79 ± 1.14 count/mm^2^, *p* < 0.05; Day 28, 296.06 ± 13.29 count/mm^2^ vs. 170.61 ± 6.70 count/mm^2^, *p* < 0.01) and also exhibited an increased M2/M1 macrophage ratio (Day 7,0.99 ± 0.06 vs. 0.81 ± 0.02, *p* < 0.05; Day 28, 1.37 ± 0.04 vs. 0.51 ± 0.04, *p* < 0.01), indicating a predominant M2 phenotype on the implant. In contrast, the control group had a lower density of CD206-positive cells than the VEGF group and a reduced M2/M1 macrophage ratio. These results can be observed in the representative images (Figures 6A, B).

**FIGURE 7:**
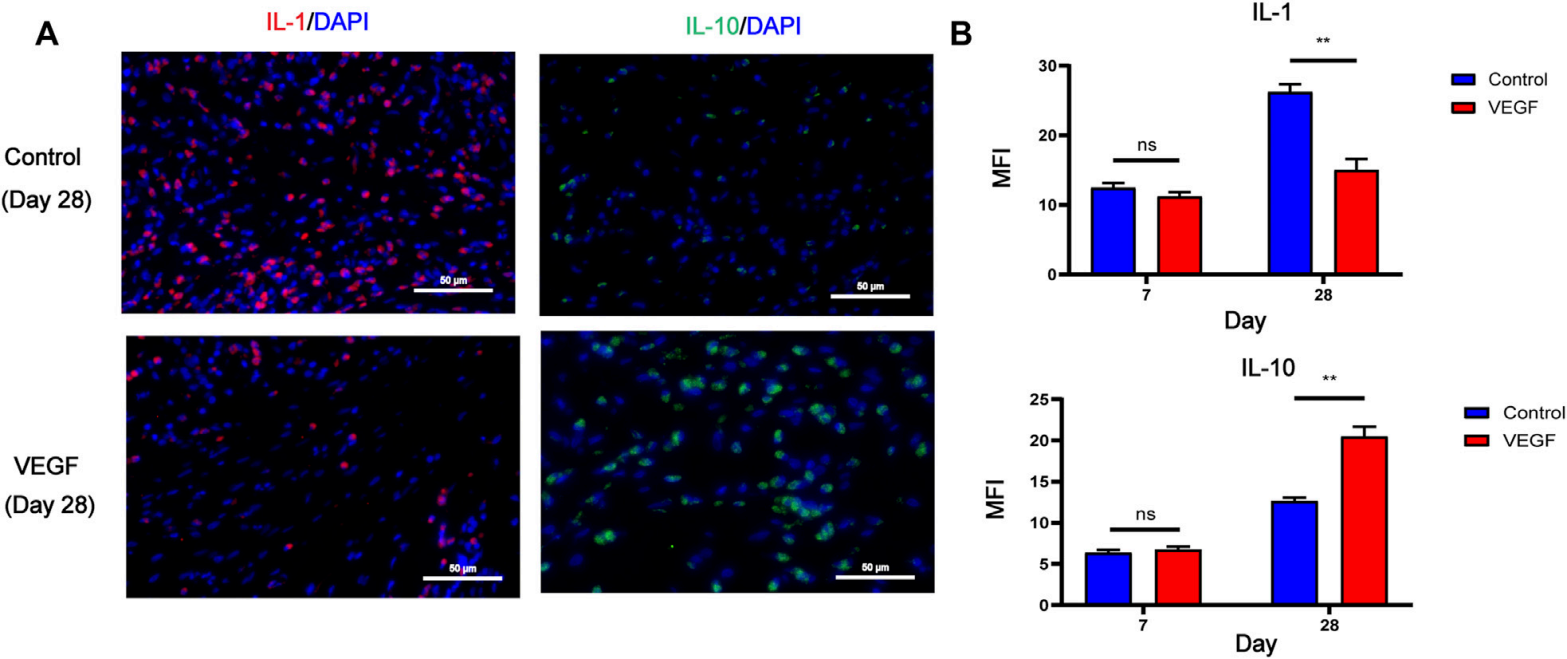
Representative immunofluorescence images of IL-1 and IL-10 expression of subcutaneous implants at day 28 in each group. IL-1 stained in red, IL-10 stained in green **(A)**. Immunofluorescence quantification of IL-1 and IL-10 **(B)**. MFI, mean fluorescence intensity. *p < 0.05. **p < 0.01. ns represents no significant difference.

To investigate local inflammatory responses, we further performed immunofluorescence analysis to confirm the expression of inflammatory cytokines. IL-10 and IL-1 were selected as typical anti-inflammatory and pro-inflammatory cytokines. Compared with the control group, the VEGF group increased the expression of IL-10 and decreased the expression of IL-1 at day 28. These results suggest that VEGF provides a powerful anti-inflammatory microenvironment for subcutaneous implants (Figures 7A, B).

### 3.4 VEGF reduces neointimal hyperplasia of decellularized vascular grafts

**FIGURE 8:**
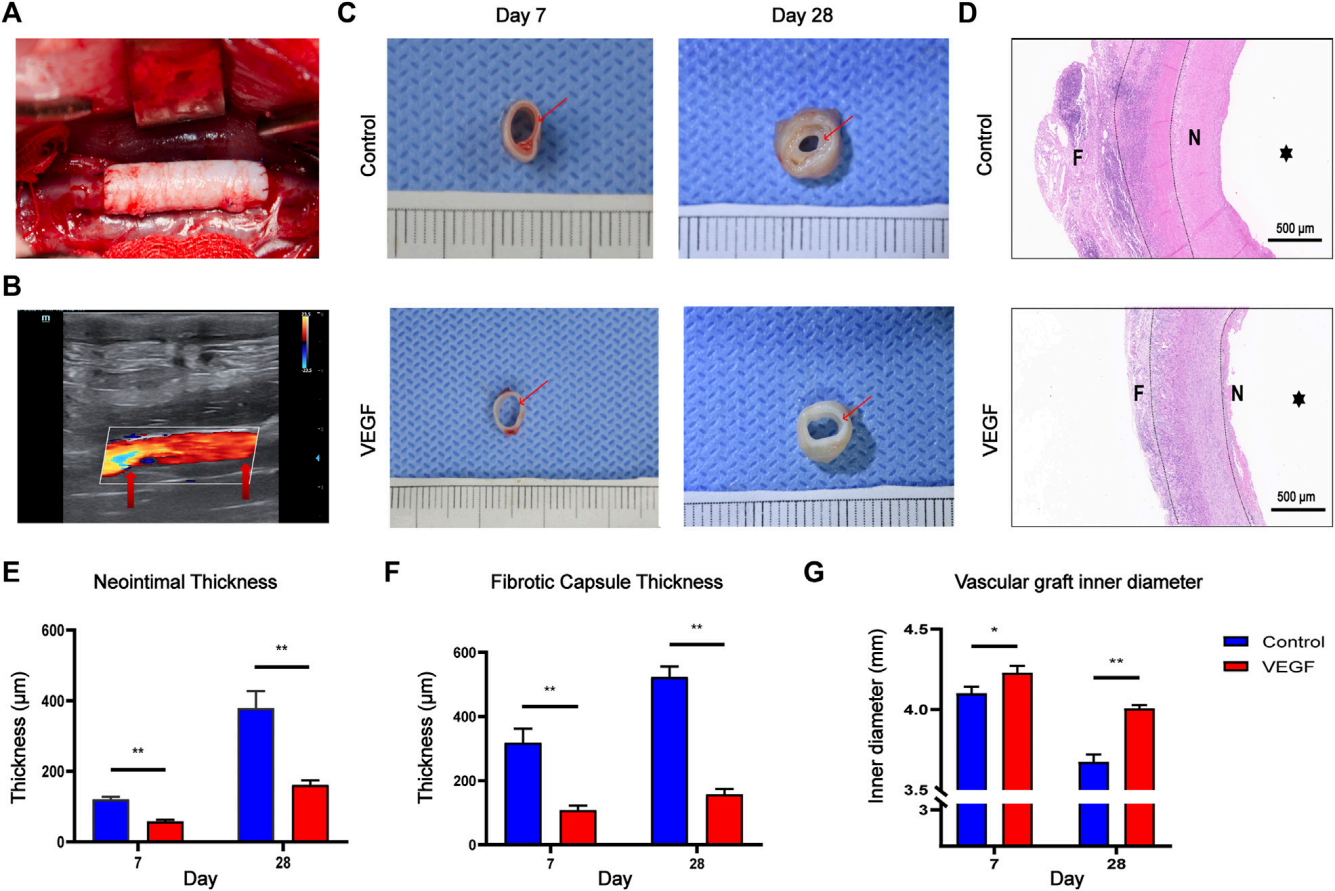
Macroscopic images of the rabbit abdominal artery implantation model **(A)**. Color Doppler ultrasound images of vascular grafts post-implantation. Red arrows depict the anastomosis **(B)**. Gross images of vascular grafts at days 7 and 28 in each group. Red arrows depict the neointima of vascular grafts **(C)**. HE staining of vascular grafts at day 28 in each group. F, fibrous capsule. N, neointima. 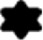, vascular lumen. Between the two dashed lines depicts the decellularized BIMA **(D)**. Measurement of neointimal thickness of vascular grafts in each group **(E)**. Measurement of vascular grafts fibrous capsule thickness **(F)**. Measurement of inner diameter of vascular grafts in each group **(G)**. *p < 0.05. **p < 0.01. ns represents no significant difference.

Small-diameter vascular grafts were implanted into the New Zealand rabbit abdominal aortic graft model and explanted at days 7 and 28 (Figure 8A). All the animals survived through the follow-up period, and the vascular grafts showed no signs of occlusion as assessed by color Doppler imaging at days 7 and 28 All animals survived during the follow-up period without paraplegia, infection and other complications. The vascular grafts in both groups showed no signs of occlusion, but there were two vascular grafts in the control group showed signs of stenosis on the 28th day as assessed by color Doppler imaging (Figure 8B, Supplementary Table S1). We observed the gross morphology of the neointima of the vascular graft, and then assessed the thickness of the neointima by HE staining (Figures 8C,D). The neointimal thickness of the vascular grafts in the VEGF group was reduced compared with the control group (Day 7, 120.57 ± 7.27 μm vs. 58.36 ± 3.94 μm, *p* < 0.01; Day 28, 379.22 ± 48.74 μm vs. 161.77 ± 13.16 μm, *p* < 0.01; Figure 8E). In addition, The fibrous capsule thickness of the grafts in the VEGF group was reduced at all time points compared to the control group (Day 7, 318.10 ± 44.39 μm vs. 108.11 ± 13.81 μm, *p* < 0.01; Day 28, 522.64 ± 33.09 μm vs. 156.92 ± 16.76 μm, *p* < 0.01; Figure 8F). The inner diameter of grafts in the VEGF group was larger than that of control group at days 7 (*p* < 0.05, Figure 8G) and 28 (*p* < 0.01, Figure 8G).

**FIGURE 9:**
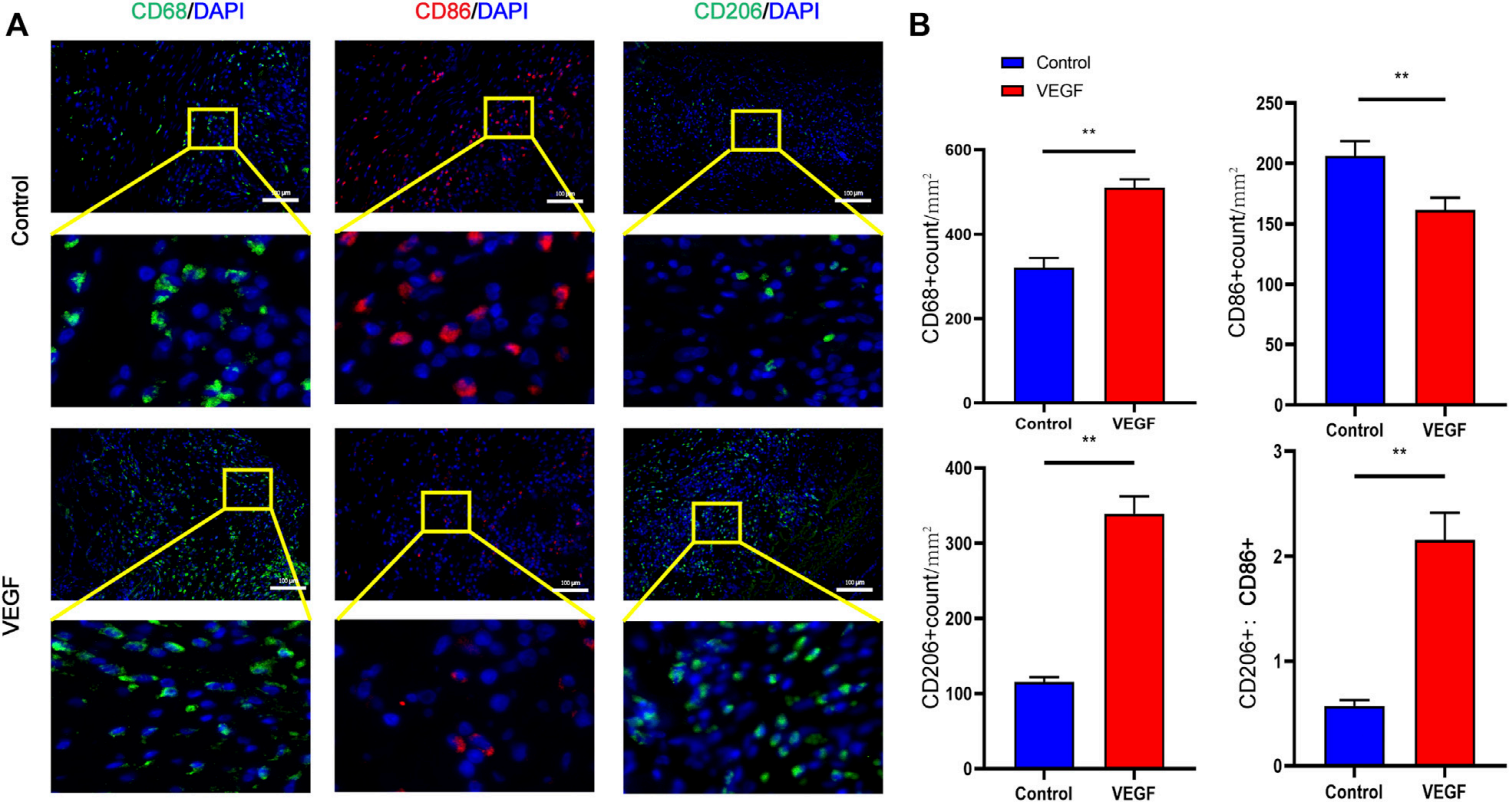
Representative immunofluorescence images of vascular grafts at day 28 in each group **(A)**. CD68 and CD206 stained in green, CD86 stained in red. Quantification of CD68^+^ total macrophages, CD86^+^ M1-macrophages, CD206+ M2-macrophages and M2/M1 (CD206+/CD86^+^) ratio **(B)**. *p < 0.05. **p < 0.01. ns represents no significant difference.

**FIGURE 10:**
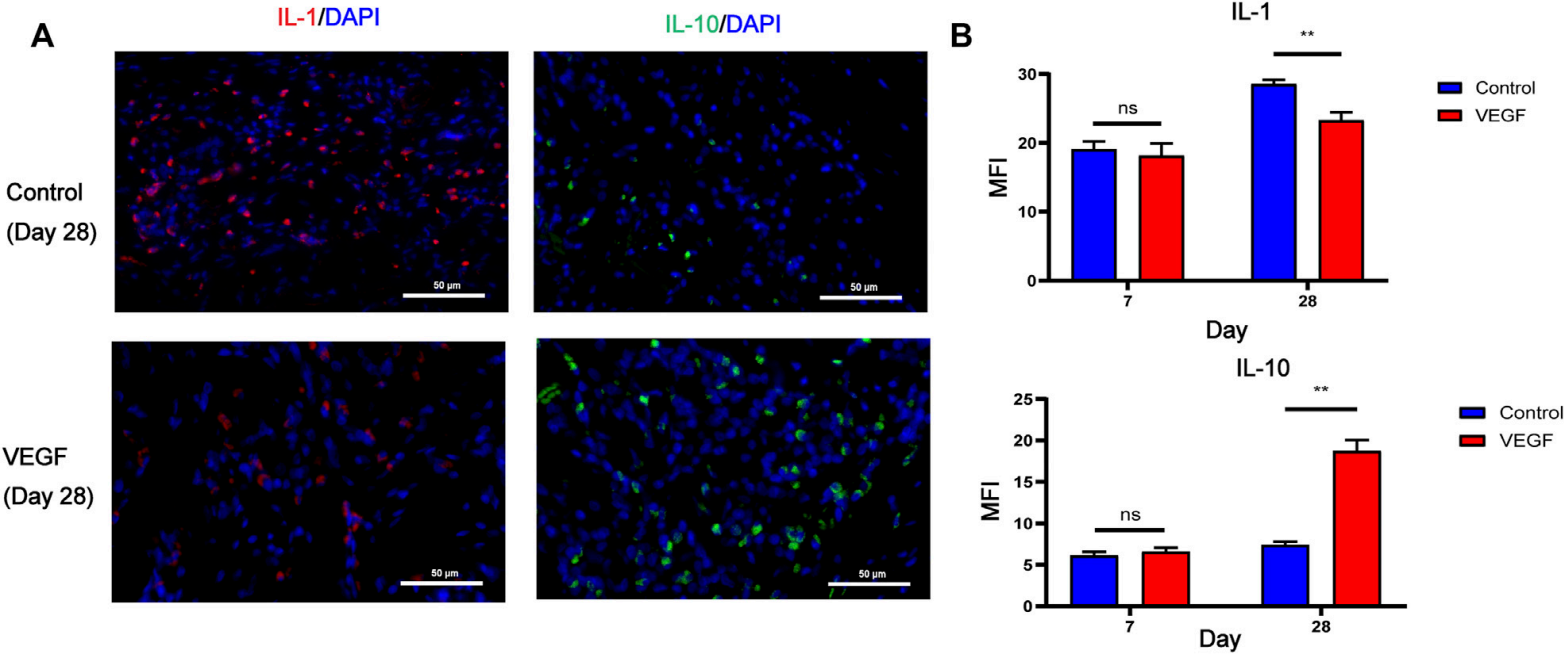
Representative immunofluorescence images of IL-1 and IL-10 expression of vascular grafts at day 28 in each group. IL-1 stained in red, IL-10 stained in green **(A)**. Immunofluorescence quantification of IL-1 and IL-10 **(B)**. MFI, mean fluorescence intensity. *p < 0.05. **p < 0.01. ns represents no significant difference.

**FIGURE 11:**
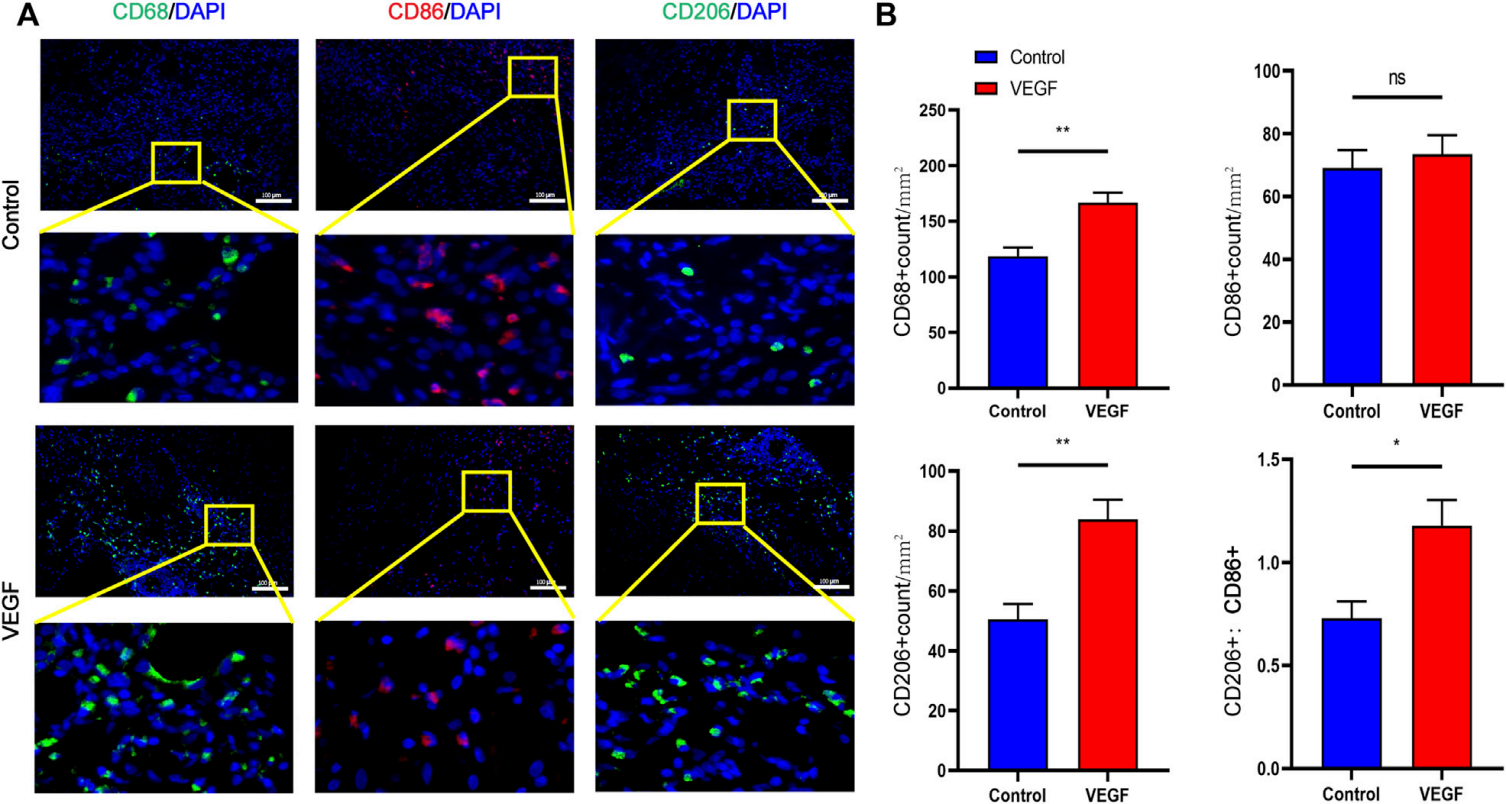
Representative immunofluorescence images of vascular grafts at day 7 in each group **(A)**. CD68 and CD206 stained in green, CD86 stained in red. Quantification of CD68^+^ total macrophages, CD86^+^ M1-macrophages, CD206+ M2-macrophages and M2/M1 (CD206+/CD86^+^) ratio **(B)**. *p < 0.05. **p < 0.01. ns represents no significant difference.

Compared with the control group, the density of total macrophages in the VEGF group was significantly increased. The increased density of CD206-positive cells (Day 7, 83.79 ± 6.64 count/mm^2^ vs. 50.50 ± 5.13 count/mm^2^, *p* < 0.01; Day 28, 338.80 ± 23.42 count/mm^2^ vs. 115.20 ± 6.70 count/mm^2^, *p* < 0.01; Figures 9, 11) and the increased M2/M1 ratio (Day 7, 1.18 ± 0.13 vs. 0.73 ± 0.08, *p* < 0.05; Day 28, 2.15 ± 0.26 vs. 0.57 ± 0.06, *p* < 0.01; Figures 9, 11) in the VEGF group compared to the control group showed a similar macrophage polarization effect. Further examination revealed an increase in anti-inflammatory IL-10 and a decrease in pro-inflammatory IL-1 expression in the VEGF group compared to the control group at day 28 (Figure 10). These results further confirmed immunomodulatory role of VEGF.

To assess the regeneration of vascular grafts, the expression of endothelial cell markers and smooth muscle cell markers of vascular grafts was analyzed. The number of eNOS-positive cells (endothelial cell markers) on the luminal surface and the density of α-SMA-positive and Calponin-positive cells (both smooth muscle cell markers) were significantly increased in the VEGF group compared to the control group at day 28 (Supplementary Figures 2A,B).

## 4 Discussion

All types of tissue engineered vascular grafts (TEVGs) will inevitably cause protein adsorption and accumulation of various inflammatory cells on the lumen surface of vascular grafts after implantation, resulting in the formation of neointima. However, excessive neointimal formation (neointimal hyperplasia) may lead to failure of vascular grafts. Xenogeneic decellularized small-diameter vascular grafts are a promising material that are readily available and their cellular components are fully removed, which can reduce biomaterial antigenicity, reduce the foreign body reaction, and is conducive to tissue regeneration. However, our previous study found that neointimal hyperplasia also was observed in xenogeneic decellularized small-diameter vascular grafts, which could decrease long-term patency of vascular grafts (Liu et al., 2022). Therefore, it is necessary to find a novel strategy to attenuates the neointimal hyperplasia of decellularized small-diameter vascular grafts.

Adequate decellularization of the ECM scaffold is the primary condition for its constructive tissue remodeling *in vivo*, which is characterized by the rapid transformation of infiltrating macrophages from M1 pro-inflammatory phenotype to M2 anti-inflammatory phenotype within a few days of implantation (Londono and Badylak, 2015). The bovine internal mammary artery has requisite length and suitable diameter, is a potential small-diameter vascular substitute, and may be used clinically in the future (Liu et al., 2022). We perform decellularization using a combination of physical, chemical and enzymatic methods to increase the efficiency of cell removal each tissue. Sodium dodecyl sulfate (SDS) and sodium deoxycholate (SDC) are ionic detergents commonly used for decellularization, and tissue engineering of SDS and SD decellularized allogeneic heart valve heart valve ECM scaffolds has been successfully used in clinical practice (Cebotari et al., 2011). Our decellularization process minimizes disruption to the structural integrity of the ECM and preserves the biomechanical properties of the xenogeneic material. We established a viable procedure for the manufacture of BMIAs ECM scaffolds based on prior literature findings and our early investigations. This protocol is based on a combination of commonly used detergents (SDS and SDC) to efficiently remove cellular components while preserving the biochemical and biomechanical properties of the scaffolds (Pu et al., 2018).

Macrophages are the main response cells in the early stage after biomaterial implantation, which have different phenotypes and are classified into M1 and M2 types (Zhang and King, 2022). M1 macrophages, also known as pro-inflammatory macrophages, secrete pro-inflammatory cytokines such as IL-1β, tumor necrosis factor alpha and IL-6, etc (Sheikh et al., 2015). M1 macrophages are thought to be involved in the synthesis of collagen, fibroblasts, and myofibroblasts, including their secretion of pro-inflammatory factors that contribute to the formation of fibrous capsules (Shin et al., 2018). M2 macrophages, also known as anti-inflammatory macrophages, are involved in repair and tissue remodeling. M2 macrophages secrete anti-inflammatory cytokines (such as IL-10) that limit the inflammatory response and contribute to tissue remodeling (Klopfleisch, 2016). The phenotype of macrophages can be switched according to the surrounding microenvironment. M1 polarization could lead to the foreign body response, resulting in dense fibrous capsule formation. M2 polarization could suppress the inflammatory response and reduce the thickness of the fibrous capsule. It is well known that VEGF is an angiogenesis stimulator that promotes endothelial cell proliferation, which can stimulate vascularization of biomaterials and endothelialization of small-diameter vascular grafts (Koobatian et al., 2016; Iijima et al., 2018; Kong et al., 2019). Therefore, VEGF is widely used in the field of tissue engineering. However, VEGF also acts as a chemokine for monocytes and macrophages, recruiting macrophages to these sites when tissue is hypoxic or inflamed (Lee et al., 2013). At the same time, several studies have found that VEGF may promote the transition of macrophages to M2 phenotype, thereby regulating the local immune microenvironment (Wheeler et al., 2018; Smith et al., 2019). Karen C. Wheeler et al. found that both endogenous and exogenous VEGF plays an important role in recruitment/migration and polarization of macrophages (Wheeler et al., 2018). At present, RAW264.7 cells are widely used to study the differentiation and function of monocytes and macrophages. In this study, our results show that VEGF may induce RAW264.7 macrophages to the M2 phenotype. We also found that increasing the concentration of VEGF can improve the polarization efficiency of RAW264.7 cells up to a maximum concentration of 50 ng/ml, but higher concentration of VEGF (100 ng/ml) inhibits the polarization efficiency (Petreaca et al., 2008; Wheeler et al., 2018). However, the precise mechanism of VEGF inducing M2 polarity in macrophages is still unclear. Macrophages express both VEGFR1/Flt1 and VEGFR2/Flk1, and VEGF may directly influence macrophage function mediated by these receptors (Wheeler et al., 2018). A. G. Ravin et al. coated Polycarbonate disks with VEGF and then implanted them into the muscle layer of Sprague–Dawley retired breeder rats. After 50 days of implantation, the fibrous capsule thickness of polycarbonate discs coated with VEGF was significantly thinner than that of those without VEGF (Ravin et al., 2001). In this study, the impact of VEGF on fibrous capsule thickness was evaluated and our results suggest that the fibrous capsule thickness of the decellularized vascular grafts was significantly reduced in the VEGF group. We employed a greater dosage of VEGF (200 ng) in the preliminary experiment and discovered that higher concentration of VEGF reduced the fibrous capsule thickness of decellularized vascular grafts, but also prevented the invasion of host cells (Supplementary Figures 1A–D). Since the absence of cell infiltration proved detrimental to tissue regeneration, we did not use this dosage in subsequent studies. Hyun-Seok Kim et al. found that inducing M2 macrophage polarization on implants might reduce pro-inflammatory factor release while increasing anti-inflammatory factor secretion, leading in a reduction in implant fibrous capsule thickness (Kim et al., 2021). In the present study, unsurprisingly, M2 macrophage polarization was observed in decellularized vascular grafts in the VEGF group. Therefore, local application of drugs to alter the immune response appears to be an effective approach to reduce fibrous capsule formation.

In the early stage, the development of neointimal formation in TEVGs is similar to the process of foreign body reaction, and both biological processes undergo protein adsorption, followed by inflammation and immune cell recruitment. Due to the lack of endothelial cells on the lumen surface, a large amount of plasma proteins will be adsorbed on the vascular graft after implantation, which is mainly determined by the hydrophobicity, topology, and surface charge of the material itself (Xu et al., 2014). Afterwards, neutrophils and monocyte/macrophages are also recruited into the neointima of the lumen by various cytokines and chemokines. In the later stage, the neointimal hyperplasia, like the fibrous capsule, contains proliferation of fibroblasts and excessive collagen deposition, which may cause vascular grafts stenosis (Chen et al., 2022). In this study, the macrophages in the control group were dominated by M1 pro-inflammatory macrophages at 7 and 28 days after implantation, indicating that persistent inflammation occurred after implantation of decellularized xenografts *in vivo*, which will lead to fibrosis or fibrous encapsulation. At 28 days, we also found apparent fibrous capsule (in subcutaneous implantation model) and neointima formation (in abdominal artery implantation model) by HE staining of decellularized vascular grafts.

Various strategies have been reported to inhibit the neointimal hyperplasia, but all of them have various drawbacks. The antiproliferative drugs paclitaxel and sirolimus can inhibit neointimal hyperplasia by inhibiting vascular smooth muscle cell proliferation, but hinder vascular endothelialization (Hayashi et al., 2009; Chaudhary et al., 2016). The clodronate inhibit neointimal hyperplasia by depleting macrophages, but prevent vascular remodeling (Hibino et al., 2011). In addition, early endothelialization of grafted vessels may inhibit neointimal hyperplasia (Lopera Higuita and Griffiths, 2020b; Zhuang et al., 2021). However, it is difficult to form an intact endothelial cell layer on the luminal surface for most vascular grafts, even after several weeks of the implantation (Zhuang et al., 2021). Regulation the immune response by local application of drugs may be a promising approach to reduce neointimal hyperplasia. Tsung-Neng Tsai et al. found that local application of VEGF reduced neointimal hyperplasia in decellularized vessel grafts at 4 weeks after implantation in mouse carotid artery replacement model. However, the authors did not clarify the mechanism by which VEGF reduces intimal hyperplasia (Tsai et al., 2012). In the present study, we demonstrated that local application of VEGF to the adventitia of decellularized vascular grafts significantly reduced their neointimal hyperplasia, which is similar to the results of Tsung-Neng Tsai et al. Tan et al. used cytokine interleukin-4 to coat electrospun polycaprolactone surfaces to create vascular grafts. These grafts repolarize macrophages to M2 anti-inflammatory phenotypes, leading to modulation of the proinflammatory microenvironment, ultimately reducing foreign body encapsulation and the development of neointimal hyperplasia (Tan et al., 2019). In this study, the effect of VEGF on neointimal thickness was assessed. Results of HE staining image analysis showed that vascular grafts with local application of VEGF had reduced neointimal thickness compared to conventional decellularized vascular grafts. The number of CD68 positive cells in the VEGF group was higher than that in the control group at days 7 and 28 in abdominal artery implantation model, but there was no significant difference in the subcutaneous implantation model. This difference may be due to the inconsistent *in vivo* environment to which the grafts are exposed, with the vascular grafts in the abdominal artery implantation model having direct contact with the circulating blood containing a large number of monocytes. This difference in the *in vivo* environment may likewise account for the inconsistency between the neointima formation process and the classical foreign body reaction process. In addition, M1/M2 macrophage polarization of vascular grafts was investigated. As expected, local application of VEGF promoted M2 macrophage polarization of the implants and decreased the expression of pro-inflammatory cytokines (IL-1) while increasing the expression of anti-inflammatory cytokines (IL-10). Thus, these results suggest that VEGF may induce macrophages in vascular graft to an M2 anti-inflammatory phenotype, leading to amelioration of the inflammatory response and ultimately inhibiting the development of neointimal hyperplasia.

## 5 Study limitations

This study focuses on the neointimal hyperplasia of small diameter vascular grafts, and the observation time was limited to 4 weeks. Therefore, we cannot evaluate the long-term patency rate of decellularized vascular grafts. In addition, endothelial cells were found on the lumen surface of vascular grafts in both groups, but they did not reach endothelialization. Therefore, in the future, we will extend the observation time to evaluate the performance of decellularized vascular graft *in vivo* and the influence of VEGF on them. The animal model used in this study is the New Zealand rabbit. Compared with rodents and rabbits, large animals such as pigs and sheep are more similar to humans in terms of coagulation system, hemodynamics and hematological profiles. Therefore, it is necessary to implant small-diameter vascular materials in larger animals to evaluate its quality and function before clinical use (Fang et al., 2021). The use of VEGF in this study is simple and effective. However, it is difficult to achieve the same effect due to different operators in clinical practice. Therefore, it is necessary to find a local drug delivery method that can achieve stable effect for clinical transformation. Injectable hydrogel has several ideal characteristics in therapeutic applications, including targeted delivery, easy repeated delivery, and polymerization to form a structure suitable for host cell infiltration (Wolf et al., 2012). Therefore, local application of VEGF loading in hydrogels to the adventitia of decellularized small-diameter vascular grafts may be a promising approach.

## 6 Conclusion

In conclusion, we decellularized BIMAs while preserving its extracellular matrix components and biomechanical properties. The bioactive molecule VEGF induces the polarization of macrophages towards their anti-inflammatory M2 phenotype, modulates the inflammatory response, resulting in a significant decrease in fibrous capsule development and neointimal hyperplasia after implantation of decellularized vascular grafts. Our findings have substantial significance for improving the inflammatory response of biomaterials and the long-term functioning of small-diameter vascular grafts.

## Data Availability

The original contributions presented in the study are included in the article/Supplementary Material, further inquiries can be directed to the corresponding author.
