# Supplementary material for: Vascular endothelial growth factor attenuates neointimal hyperplasia of decellularized small-diameter vascular grafts by modulating the local inflammatory response
Source: Front Bioeng Biotechnol. 2022 Dec 20;10:1066266. doi: 10.3389/fbioe.2022.1066266 (PMC9808043; doi:10.3389/fbioe.2022.1066266)
Supplement: Supplementary file 1 [file DataSheet1.docx]

Table S1: Summary for abdominal aorta replacement in rabbit (n = 5, per group per time point).

|  | 7 day | | | 28 day | | |
| --- | --- | --- | --- | --- | --- | --- |
|  | Occlude | Stenosis | Patency (%) | Occlude | Stenosis | Patency (%) |
| Control | 0 | 0 | 100 | 0 | 2 | 100 |
| VEGF | 0 | 0 | 100 | 0 | 0 | 100 |

Supplemental Fig.1:


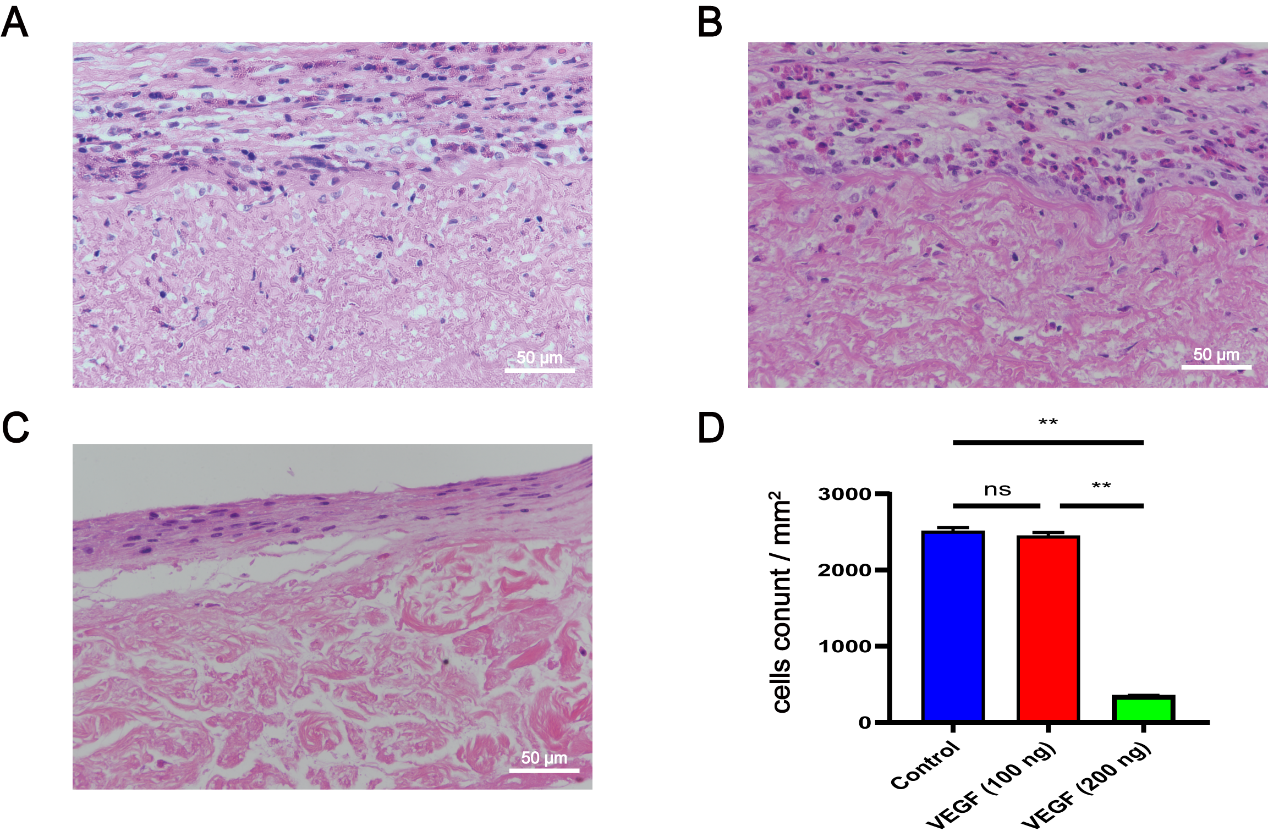


Supplemental FIGURE.1. In vivo recellularization of decellularized vascular grafts in rabbit subcutaneous implantation model at day 28. Representative HE staining of subcutaneous implants in control group (A). Representative HE staining of subcutaneous implants in VEGF group (dosage,100 ng) (B). Representative HE staining of subcutaneous implants in VEGF group (dosage,200 ng) (C). Quantification of cell infiltration of subcutaneous implants in each group at day 28 (D). *P< 0.05. **P< 0.01. ns represents no significant difference.

Supplemental Fig.2:


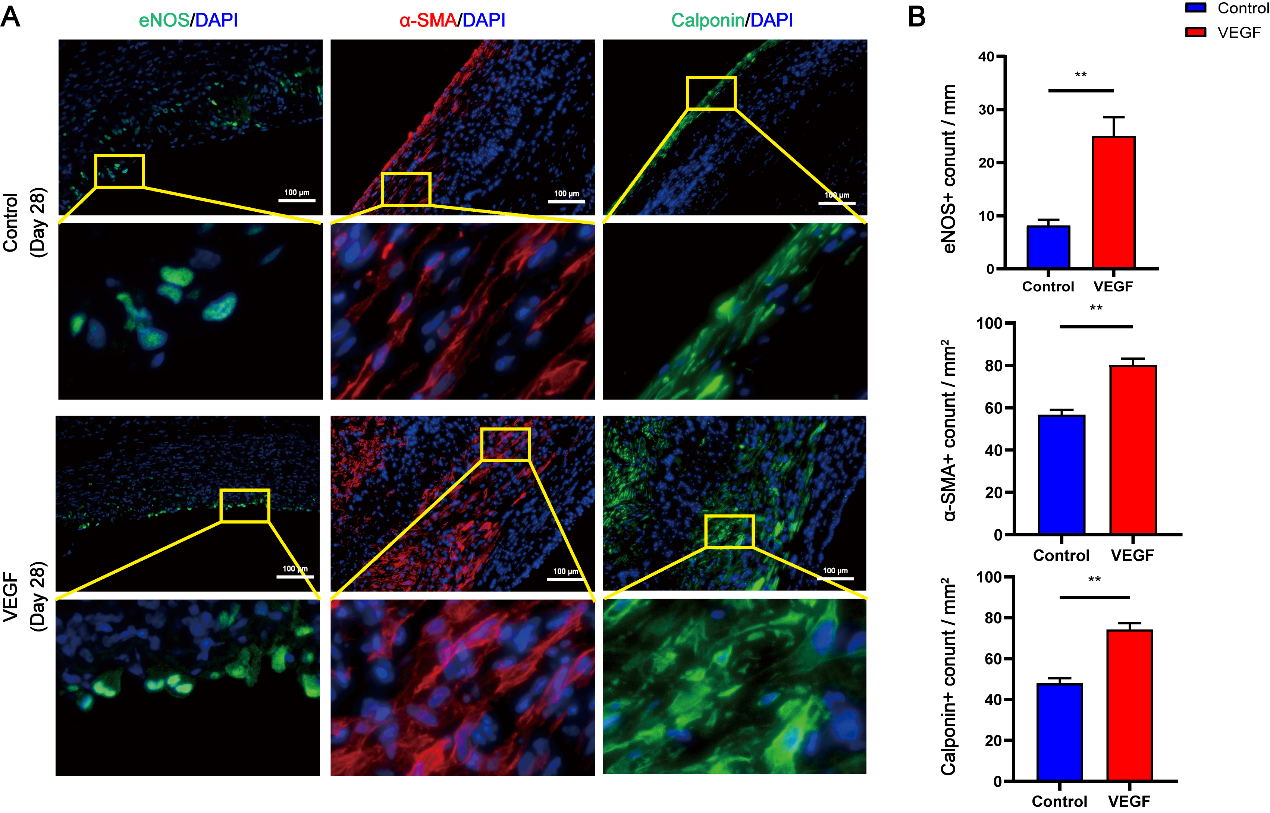


Supplemental FIGURE.2. Regeneration of endothelial cells and smooth muscle cells in rabbit abdominal artery implantation model. Representative immunofluorescence images of vascular grafts at day 28 in each group (A). eNOS and Calponin stained in green, α-SMA stained in red. Quantification of eNOS + endothelial cells, α-SMA + smooth muscle cells and Calponin + smooth muscle cells (B). *P < 0.05. **P < 0.01. ns represents no significant difference.
